# The Tariff on Instruments, Books, and Drugs

**Published:** 1888-08

**Authors:** 


### Editorial, Etc.

---

THE TARIFF ON INSTRUMENTS, BOOKS, AND DRUGS.—The New York Neurological Society has refused to indorse the resolution of the Georgia Medical Society requesting the removal of the tariff on medical books, surgical instruments, and drugs. As the Neurological Society contains a large number of believers in tariff reduction, their action must have been dictated by the best interests of the profession. It is easy to see on what grounds they opposed this resolution, originally offered as a piece of buncombe merely. While a tax remains on the raw material the removal of the tariff on instruments discriminates against the American instrument-maker, and must ultimately raise the price of instruments. Furthermore, under existing circumstances, a surgeon who desires an instrument can have it made by a maker at his door. If the tariff on instruments be swept away without removing the tariff on raw material, the instrument-maker's trade is destroyed, and the surgeon must import his instruments. The fact that the members of the New York Society are greater importers of books and instruments than those of the Georgia Medical Society, renders their action peculiarly significant.—*Medical Standard.*

As off-setting this action of The New York Neurological Society, the Medical Society of the County of New York passed resolutions urging upon Congress the repeal of the import duty on medicines, medical and surgical appliances, and everything used in the treatment and diagnosis of disease. Attention was called to this action of the Medical Society of the County of New York in *The Journal* OF April 7, though the *Medical Standard* seems to have found it convenient to overlook what the larger and more important Society, composed of general practitioners as well as specialists, has done. While the *Standard* claims that it is easy to see on what grounds the Neurological Society opposed the resolution of the Georgia State Medical Society, it, however, has failed to see what is so visible. The N. Y. Neurological Society took the ground, when it refused to endorse the resolutions of the Georgia Society, that the charges against native instrument-makers were too intemperate and sweeping. The Neurological Society said nothing about "discrimination against the American instrument-maker." Nor did the Neurological Society advance the entirely original and very unique opinion that the removal of the tariff on instruments would "ultimately raise the price of instruments."

When the *Medical Standard* says that the resolutions of the Georgia Medical Society were "originally offered as a piece of buncombe merely," it offered a gratuitous, unmerited, and entirely uncalled for insult to a scientific body of gentlemen whose time and lives are being spent in the interests of humanity. As medical men what have we to do with the instrument-makers' trade as compared with the health, comfort, and lives of sick people? Admitting that the members of the Neurological Society import more books and instruments than those of the Georgia Medical Society, do they import more than the members of the New York County Medical Society? Again, if the action of the Neurological Society "must have been dictated by the best interests of the profession," why did the members reject that portion of the resolutions referring to books and drugs, because the charges against instrument-makers were too intemperate and sweeping? Instrument-makers do not import drugs and books.

In this connection we beg to call attention to the following, from the *Medical Record* of July 28: In a speech on tariff reform delivered by Hon. Ashbel P. Fitch, of New York; a letter was read from a New York pathologist, in which he said among other things: "For my microscope I sent to Jena, where are made the best instruments for my work. At the factory it cost $94; to get it out of the custom-house 40 per cent more. Later I sent for an oil immersion lens, and paid $80 at the factory, 40 per cent. more at the custom-house. Herman Katsch, of Berlin, makes an instrument called microtome, for cutting infinitely thin sections or shavings from the surface of a piece of an organ of the body, hardened in alcohol. Herr Katsch is the only man in the world who makes this particular variety of the instrument. To prepare a section thin enough for careful study under the high powers of the microscope this mechanism is necessary. To get this microtome from the custom-house I had to wait two weeks and pay a duty of 40 per cent. on its factory price. The celebrated Dr. Koch, of Berlin, published a report of the cholera commission, conducted under the auspices of the Government. At most, twenty men in this country could require this work, and they must needs pay 25 per cent. duty to get it from the custom-house after paying its publisher's price and freight. What use could this report be to these scientists? To aid them in maturing methods of recognizing the disease when it appeared on shipboard in our harbors; to devise means to suppress it, to protect the country. It was to the expert work of one such scientist that the city of New York must give its gratitude that a certain steamship just developing cholera among its steerage passengers was detained at quarantine and the city escaped overwhelming infection. For Koch's report he paid 25 per cent. duty, and never received anything from the city or Government. When we look up from our laboratory tables, microscopes, microtomes, and alcohol—taxed to suffocation—and read in the papers of the United States Treasury filled to suffocation, we reflect that our scientific work takes much time, brings no money return, increases our outgoes, and has not even the encouragement of the Government nor laity.—*Journal of American Medical Association.*

